# Myocardial T1, T2, T2*, and fat fraction quantification via low‐rank motion‐corrected cardiac MR fingerprinting

**DOI:** 10.1002/mrm.29171

**Published:** 2022-01-26

**Authors:** Gastao José Lima da Cruz, Carlos Velasco, Begoña Lavin, Olivier Jaubert, Rene Michael Botnar, Claudia Prieto

**Affiliations:** ^1^ School of Biomedical Engineering and Imaging Sciences King’s College London London United Kingdom; ^2^ Department of Biochemistry and Molecular Biology School of Chemistry Complutense University Madrid Spain; ^3^ Escuela de Ingeniería Pontificia Universidad Católica de Chile Santiago Chile

**Keywords:** cardiac MR, fat fraction, low rank, motion correction, MR fingerprinting, T1 mapping, T2 mapping, T2* mapping

## Abstract

**Purpose:**

Develop a novel 2D cardiac MR fingerprinting (MRF) approach to enable simultaneous T1, T2, T2*, and fat fraction (FF) myocardial tissue characterization in a single breath‐hold scan.

**Methods:**

Simultaneous, co‐registered, multi‐parametric mapping of T1, T2, and FF has been recently achieved with cardiac MRF. Here, we further incorporate T2* quantification within this approach, enabling simultaneous T1, T2, T2*, and FF myocardial tissue characterization in a single breath‐hold scan. T2* quantification is achieved with an eight‐echo readout that requires a long cardiac acquisition window. A novel low‐rank motion‐corrected (LRMC) reconstruction is exploited to correct for cardiac motion within the long acquisition window. The proposed T1/T2/T2*/FF cardiac MRF was evaluated in phantom and in 10 healthy subjects in comparison to conventional mapping techniques.

**Results:**

The proposed approach achieved high quality parametric mapping of T1, T2, T2*, and FF with corresponding normalized RMS error (RMSE) T1 = 5.9%, T2 = 9.6% (T2 values <100 ms), T2* = 3.3% (T2* values <100 ms), and FF = 0.8% observed in phantom scans. In vivo, the proposed approach produced higher left‐ventricular myocardial T1 values than MOLLI (1148 vs 1056 ms), lower T2 values than T2‐GraSE (42.8 vs 50.6 ms), lower T2* values than eight‐echo gradient echo (GRE) (35.0 vs 39.4 ms), and higher FF values than six‐echo GRE (0.8 vs 0.3 %) reference techniques. The proposed approach achieved considerable reduction in motion artifacts compared to cardiac MRF without motion correction, improved spatial uniformity, and statistically higher apparent precision relative to conventional mapping for all parameters.

**Conclusion:**

The proposed cardiac MRF approach enables simultaneous, co‐registered mapping of T1, T2, T2*, and FF in a single breath‐hold for comprehensive myocardial tissue characterization, achieving higher apparent precision than conventional methods.

## 1 INTRODUCTION

Myocardial tissue characterization has become an important adjunct of clinical diagnosis in patients with heart disease over the past decade due to advances in quantitative MRI (qMRI). In contrast to qualitative imaging, qMRI promises more accurate, reproducible, and objective classification of both focal and diffuse disease. Measurement of relaxation parameters such as T1, T2, T2*, and extracellular volume (ECV) are recommended by the Society for Cardiovascular Magnetic Resonance (SCMR)^1^ for several conditions. Different relaxation parameters are useful biomarkers for different diseases. Native T1, together with ECV, are used to characterize infarction, amyloidosis, and fibrosis, among others.^2^ There is cumulating evidence of the importance of T2 mapping in detecting and quantifying myocardial edema, acute infarction, inflammation, or transplant rejection.^1^T2* is particularly relevant in detection and follow‐up of iron overload, but can also help in cases of necrosis or hemorrhage.^3^ Additionally, fat fraction (FF) quantification is important to detect fatty infiltrations as well as evaluate epicardial fat.^4^, ^5^ Due to the complementary information these parameters provide multi‐parametric cardiovascular MR (CMR) imaging is desirable for comprehensive disease characterization.

Single parameter mapping techniques are commonly used in clinical and research settings, including MOLLI^6^ and SASHA^7^ for T1, T2prep‐bSSFP^8^ and T2‐GraSE^9^ for T2, multi‐echo gradient echo (GRE) for T2*,^10^, ^11^ and multi‐echo GRE for water/fat separation and/or FF quantification.^4^ Recent research has focused on multi‐parametric mapping (primarily on simultaneous T1 and T2) from a single scan, resulting in novel frameworks like 3D‐QALAS,^12^ CABIRIA,^13^ T1/T2 combining MOLLI, and T2prep‐bSSFP ideas^14^ or combining SASHA and T2prep‐bSSFP ideas,^15^ among others.^16^, ^17^, ^18^ These methods generally rely on steady‐state signal models that are combined with exponential fitting or Bloch‐equation/Extended Phase Graph (EPG)^19^, ^20^ dictionary based matching to retrieve the underlying relaxation times.

MR fingerprinting (MRF)^21^ is an alternative framework for multi‐parametric mapping that aims to extract MR parameters from the continuous transient‐state signal. In MRF, sequence parameters (e.g., flip angle and TR) are varied to estimate the underlying tissue parameters (e.g., T1 and T2). Bloch simulations are used to predict the entire signal response of the sequence (known as a fingerprint); instead of exponential fit models, the acquired fingerprints are compared to a pre‐simulated list of fingerprints (known as a dictionary) to identify the best match, and consequently the underlying T1 and T2 values. MRF is considered here as any method that: (1) resolves the transient state magnetization for the purposes of (multi‐) parametric mapping, (2) uses dictionaries to capture the MR physics information of a given sequence and estimate its underlying parameters (as opposed to fitting routines), and (3) uses incoherent (spatial and parametric) encoding patterns to enable highly undersampled acquisition schemes.

MRF was initially extended to 2D cardiac imaging, enabling simultaneous T1 and T2 mapping.^22^ The encoding of T1 and T2 is performed via inversion recovery (IR) pulses (similar to MOLLI) and T2‐preparation pulses (similar to T2prep‐bSSFP), using electrocardiogram (ECG) triggering and breath‐holds to deal with cardiac and respiratory motion, respectively. A 3D cardiac MRF for T1/T2 has been developed,^23^ following a similar strategy but incorporating respiratory motion correction to enable free‐breathing scans. Simultaneous mapping of T1/T2 together with CINE imaging has also been developed with free‐running 2D cardiac MRF.^24^, ^25^ In addition to T1 and T2, FF has recently been incorporated into the simultaneous multi‐parametric mapping using 2D cardiac MRF.^26^, ^27^

In this work, we further incorporate T2* quantification within an extended 2D cardiac MRF approach, enabling simultaneous T1, T2, T2*, and FF myocardial tissue characterization in a single breath‐hold scan. This approach enables simultaneous mapping of three SCMR recommended quantitative parameters^1^ in a single scan, in addition to FF mapping. For the patient, this approach enables less breath‐holds and less scan time, improving comfort and experience. For the radiographer, this means less scans to plan, also improving patient throughput. For the cardiologist, this approach produces four co‐registered parametric maps, greatly facilitating analysis, as all maps are obtained at exactly the same cardiac and respiratory motion state. In the proposed sequence, T1 and T2 are encoded with IR and T2prep pulses; whereas FF and T2* encoding is based on eight‐echo GRE readouts, which significantly increases the acquisition window of the existing cardiac MRF sequences, from ~150–200 ms to ~500 ms. A recently proposed low‐rank motion‐corrected (LRMC) reconstruction^28^ is exploited to correct for the cardiac motion within the extended acquisition window. Low‐dimensional subspace constrained reconstructions have enabled highly accelerated parameter mapping techniques.^29^, ^30^, ^31^, ^32^, ^33^, ^34^, ^35^, ^36^, ^37^, ^38^, ^39^ However, these low‐rank (matrix) approaches do not explicitly model elastic motion in the encoding operator, which limits the effectiveness of such low‐rank models in the presence of motion.^40^ A recent MRF study^25^ has incorporated image‐based motion correction by warping the reconstructed singular images to a common reference motion state prior to dictionary matching. Here, we proposed a reconstruction‐based approach that incorporates the motion correction directly into the reconstruction process. Generalized motion corrected reconstructions^41^, ^42^, ^43^, ^44^, ^45^ have been developed for single contrast imaging. This formulism, introduced by Batchelor et al,^41^ incorporates dense motion fields (i.e., one displacement vector per pixel, per motion state) into the encoding operator. However, existing formulations do not consider dynamic contrast as in the case of MRF. LRMC performs generalized motion correction within a low‐rank subspace to correct for cardiac motion and resolve the dynamically changing contrasts in the proposed highly accelerated, large cardiac window T1/T2/T2*/FF cardiac MRF. In this study, we set out to: (1) investigate the feasibility of T1/T2/T2*/FF mapping from a single breath‐held, long cardiac acquisition window MRF with and without cardiac motion correction; (2) compare the proposed T1/T2/T2*/FF cardiac MRF with reference measurements in a phantom to evaluate accuracy and precision; (3) compare the proposed T1/T2/T2*/FF cardiac MRF with conventional clinical mapping sequences in healthy subjects to access in vivo feasibility. The proposed framework was evaluated in a standardized T1/T2 phantom, custom fat phantom, and in 10 healthy subjects, in comparison to corresponding spin‐echo (phantom) and clinical reference methods. To the best of our knowledge, this is the first demonstration of simultaneous, co‐registered T1, T2, T2*, and FF myocardial MRF in a single breath‐held scan.

## 2 METHODS

### 2.1 Framework overview

**FIGURE 1:**
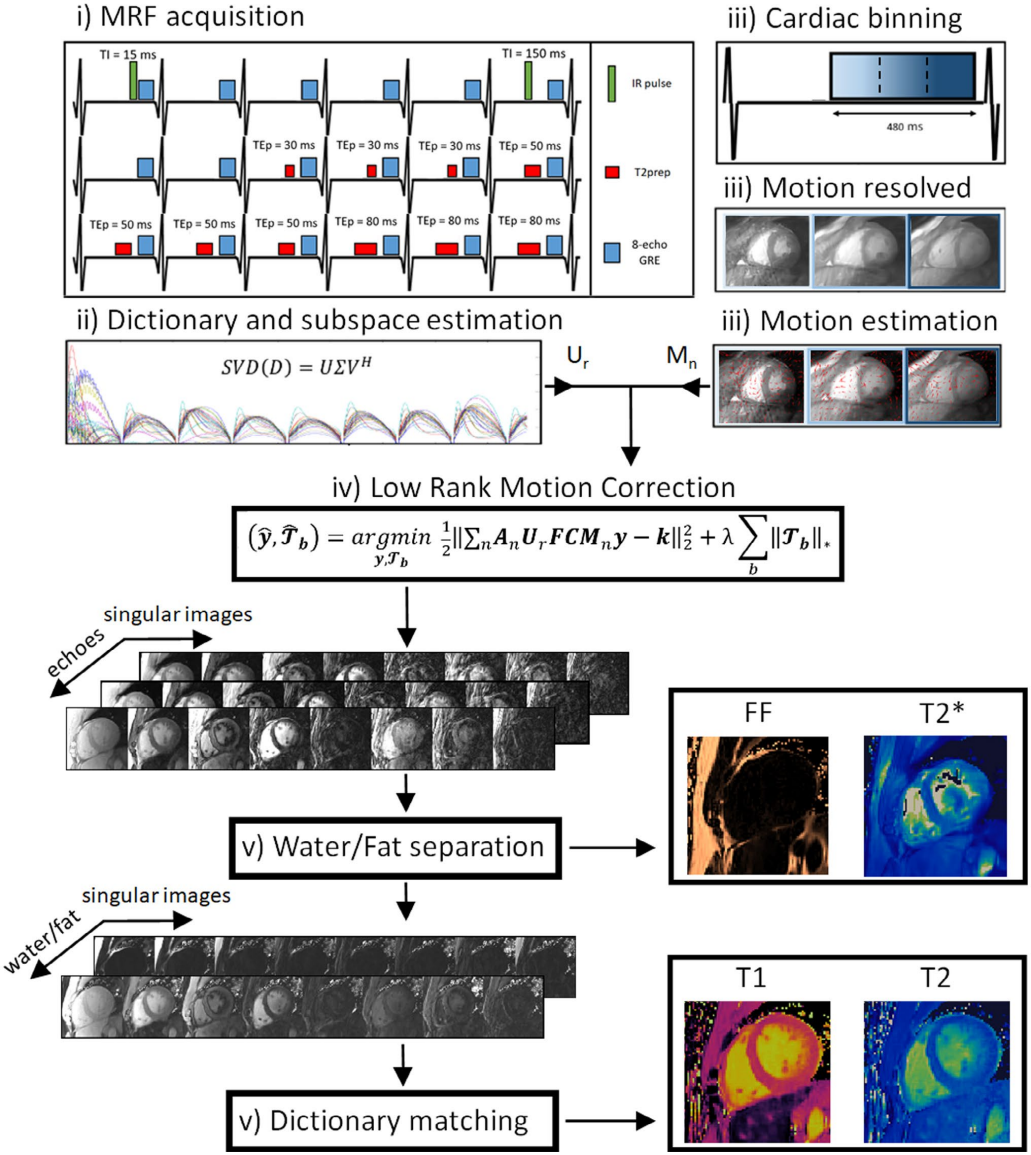
Proposed framework for simultaneous T1/T2/T2*/FF myocardial MRF. (1) A cardiac triggered acquisition with IR, T2 preparation (T2prep) pulses, and eight‐echo GRE readouts is used to encode the parameters. (2) The corresponding MRF dictionary is simulated and low‐rank basis (U_r_) determined via SVD. (3) Cardiac binning separates the long window (480 ms) data into multiple motion states; auxiliary motion‐resolved reconstructions are performed and cardiac motion fields (M_n_) estimated via image registration. (4) LRMC reconstruction is performed, producing a set of motion corrected singular images for each echo. (5) The set of first singular images is used for water/fat separation, producing a set of water/fat separated singular images, as well as FF and T2* maps; the set of water singular images is used for dictionary matching to estimate T1 and T2 maps

The proposed framework is divided in five steps (Figure 1): (1) MRF acquisition; (2) dictionary computation and subspace estimation; (3) auxiliary cardiac‐resolved reconstruction and cardiac motion estimation; (4) LRMC reconstruction; (5) water/fat separation and MRF dictionary matching (Figure 1).

In the first step, data are acquired in a breath‐held, cardiac triggered sequence over 18 heartbeats. IR preparation pulses are used to encode T1, T2 preparation (T2prep) pulses are used to encode T2, and eight‐echo GRE readouts are used to encode T2* and water/fat separation. In order to acquire sufficient data in a single breath‐hold, the cardiac acquisition window is extended to ~480 ms, considerably longer than the conventional ~150–200 ms window, thus, making it susceptible to cardiac motion. In the second step, the MRF dictionary is computed for a set of T1 and T2 values, taking into account the patient specific sequence timings (i.e., ECG triggers). A singular value decomposition (SVD) is performed on this dictionary to estimate a lower dimensional subspace for reconstruction. In the third step, data are binned into multiple cardiac phases to produce an auxiliary motion‐resolved reconstruction. These intermediate images are used to estimate the non‐rigid cardiac motion that occurs within the ~480 ms acquisition window via an image registration algorithm. In the fourth step, an LRMC^28^ reconstruction is performed, correcting for cardiac motion in the dictionary‐derived low‐rank basis, simultaneously removing motion artifacts and suppressing aliasing and noise originating from the high undersampling factors used in the proposed cardiac MRF. In the final fifth step, the first singular image of the LRMC reconstruction is used to perform water/fat separation, along with T2* and FF estimation. The resulting water separated singular images are used to estimate T1 and T2 via conventional MRF dictionary matching.

### 2.2 MRF acquisition

The proposed cardiac MRF sequence for simultaneous T1, T2, T2*, and FF is a breath‐held, ECG‐triggered sequence with duration of 18 heartbeats (Figure 1). Sequence design is based on previous cardiac MRF,^23^ using fixed, low flip angles of 15° and fixed TE and TR. The initial eight heartbeats are reminiscent of the MOLLI sequence,^46^ relying on the T1 recovery over several heartbeats following an IR pulse with varying inversion time delay (TI). Specifically, two IR pulses were considered at heartbeats #1 and #6 with corresponding TIs of 15 ms and 100 ms. The final 10 heartbeats are similar to the strategy used in T2prep bSSFP^47^ without recovery heartbeats, relying on T2preps with varying TEs to encode T2. Namely, heartbeats #9 through #18 used the following T2prep encoding scheme: 30‐30‐30‐50‐50‐50‐50‐80‐80‐80, where each number denotes the T2prep TE (TEp) for a given heartbeat. To enable T2* and FF estimation, we used a radial eight‐echo GRE bipolar readout in every heartbeat. Therefore, whereas T1 and T2 encoding are somewhat localized in time, T2* and FF encoding occurs in every heartbeat. The readout used requires longer TRs, which increases the cardiac acquisition window while maintaining a sufficient number of readouts per echo. Thirty readouts (each with eight echoes) were considered here, comparable to other cardiac MRF approaches^22^, ^23^, ^26^ (using 48, 25, and 30 readouts, respectively). Therefore, the duration of the acquisition window is extended from the conventional ~150–200 ms to ~480 ms, increasing scan efficiency and allowing T2* and FF mapping as well as T1 and T2 mapping.

### 2.3 Dictionary computation and subspace estimation

MRF dictionary computation is performed via EPG^19^, ^20^ considering an ideal slice profile, fixed B0 and fixed B1. As previously shown,^48^ errors arising from imperfect slice profile or B1 inhomogeneities are minimal when low flip angles are considered, as they are in this work. A single TE is simulated in the EPG using fixed B0 as proposed previously^26^; the MRF component of the framework estimates T1 and T2 after T2*, B0 and water/fat signals have been resolved via conventional water/fat separation methods. ECG patient‐specific signals are used to generate a patient‐specific dictionary as is common in triggered cardiac MRF. Estimation of the low‐rank subspace^29^, ^30^, ^31^, ^32^, ^33^, ^34^, ^35^, ^36^, ^37^, ^38^, ^39^ is attained via an SVD of the MRF dictionary as outlined previously.^33^ The rank value *r* is determined by the minimum value that captures 98% of the matrix energy ratio.^33^

### 2.4 Auxiliary cardiac‐resolved reconstruction and motion estimation

The LRMC reconstruction requires prior knowledge of the dense motion fields that describe the underlying cardiac motion. A single set of coil maps was estimated by combining all the acquired data via ESPIRiT.^49^ In this framework, cardiac motion is estimated from the acquired data itself, through auxiliary motion‐resolved reconstructions. The acquired data are equally divided into 10 cardiac phases with equal size of ~48 ms. The corresponding motion‐resolved images are obtained via the following reconstruction:

(1) $$\begin{array}{c} {\hat{y}}_{n},{\hat{T}}_{b}^{n}=\underset{y_{n},T_{b}^{n}}{\mathrm{argmin}}\frac{1}{2}{∥W_{n}\left(A_{n}U_{r}{\mathrm{FCy}}_{n}‐k_{n}\right)∥}_{2}^{2}+\lambda \sum_{b}{∥T_{b}^{n}∥}_{*}\\ s.t.T_{b}^{n}=Q_{b}\left(y_{n}\right) \end{array}$$

where $y_{n}$ are the reconstructed singular images for the *n‐th* motion state (or bin), $W_{n}$ are soft‐weights, $A_{n}$ corresponds to k‐space sampling, $U_{r}$ captures the signal subspace, decompressing from *r* singular images to the time domain (whereas ${U_{r}}^{H}$ compresses from time to *r* singular images), F is the non‐uniform Fourier transform, C are the coil sensitivities and $k_{n}$ is the k‐space for the *n‐th* bin. HD‐PROST^50^ regularization is used where $Q_{b}$ generates a 3D tensor $T_{b}^{n}$ of voxels associated with the *b‐th* voxel (and *n‐th* bin) by concatenating local voxels (within a local patch) along the first dimension, non‐local voxels (from patches that exhibit structural similarity with the patch around *b*) and contrast voxels (along the compressed singular value domain). To achieve improved contrast for image registration, only the heartbeats with T2preps (#9–18) are considered for auxiliary motion‐resolved reconstructions. Here, the first singular image was selected to produce the set of cardiac‐resolved images for motion estimation due to its high SNR and reduced aliasing artifacts. The resulting cardiac‐resolved images are averaged over all eight echoes to produce one image per motion state. Finally, these images are registered via intensity‐based free‐form deformations (NiftyReg)^51^, ^52^ to estimate the underlying cardiac motion. The tenth phase of the acquisition window (typically in mid‐late diastole) was chosen as the reference motion state.

### 2.5 LRMC

LRMC corrects for generalized motion while exploiting redundant information along the contrast dimension via a low‐dimensional subspace estimated from the MRF dictionary. Subspace‐based reconstructions have been proposed for several applications with varying formulations.^29^, ^30^, ^31^, ^32^, ^33^, ^34^, ^35^, ^36^, ^37^, ^38^, ^39^ Generally, they solve for a set of coefficients of some low‐rank basis (referred here as singular images) instead of solving for every image in the time‐series, which has better condition properties. LRMC reconstruction further considers generalized motion correction into the forward model, allowing us to exploit these redundancies even in the presence of arbitrary motion. The LRMC is formulated as:

(2) $$\begin{array}{c} \left(\hat{y},{\hat{T}}_{b}\right)=\underset{y,T_{b}}{\mathrm{argmin}}\frac{1}{2}{∥\sum_{n}A_{n}U_{r}{\mathrm{FCM}}_{n}y‐k∥}_{2}^{2}+\lambda \sum_{b}{∥T_{b}∥}_{*}\\ s.t.T_{b}=Q_{b}\left(y\right) \end{array}$$

where y are motion corrected singular images, $A_{n}$ is the k‐space sampling for the *n‐th* motion state, k is the acquired (motion corrupted) k‐space and $M_{n}$ is a sparse matrix that encodes the motion transformation for the *n‐th* motion state. As before, we consider HD‐PROST regularization to suppress residual aliasing and noise amplification.

The auxiliary cardiac‐resolved and the LRMC reconstructions were solved with the Alternating Direction Method of Multipliers (ADMM)^53^; the Conjugate Gradient (CG)^54^ was used to solve the L2‐regularized problems within the ADMM problem; high order SVD (HOSVD)^55^ followed by singular value thresholding was used to minimize the nuclear norm within the HD‐PROST regularized ADMM problem. Coil maps were estimated from the entire MRF dataset.^56^

### 2.6 Water/Fat separation and dictionary matching

The LRMC reconstruction produces y, a set of motion corrected singular images (for every echo). In line with our previous framework for Dixon cardiac MRF^26^ and multi‐parametric liver MRF,^57^ water/fat separation and T2* are estimated from the first singular image using a robust method by Hernando et al,^58^ considering a six‐peak fat model^59^ with fixed T1/T2 values. The resulting water and fat separated singular images are used in a conventional MRF inner product matching to recover water/fat‐specific T1, T2 and (apparent) M0. The proton density FF is estimated via the water and fat estimated M0 via the IDEAL method.^60^ The water/fat separation methods used here are available in the ISMRM Water/Fat toolbox.

### 2.7 Experiments

The proposed cardiac T1/T2/T2*/FF approach was evaluated in a standardized T1/T2 phantom “T1MES”,^61^ a custom built water/fat phantom (8 vials with FF ranging from 0–100%) in two separate scans to investigate repeatability, and in 10 healthy subjects (age = 31 ± 4 years, 7 females) at 1.5T (Ingenia, Philips, Best, The Netherlands) using a 28‐channel cardiac coil. The study was approved by the institutional review board and written informed consent was obtained from all subjects according to institutional guidelines. The proposed T1/T2/T2*/FF cardiac MRF acquisition used the following parameters: FOV = 256 × 256 mm^2^; 8 mm slice thickness; resolution = 2 × 2 mm^2^; TE_1_/ΔTE/TR = 1.6/1.8/16 ms; radial eight‐echo GRE bipolar readout; flip angle 15°; cardiac window = 480 ms; 540 time‐points; nominal scan time 18 s.

### 2.8 Phantom study

In phantom, MRF was compared against inversion recovery spin echo (IRSE) for T1, multi‐echo spin‐echo (MESE) for T2, eight‐echo GRE for T2*, and six‐echo GRE for FF. Key parameters for the IRSE included: TE/TR = 15/15000 ms, 15 TIs in the range of 50 to 5000 ms; key parameters for MESE included eight TEs in the range of 8 to 80 ms; key parameters for T2* reference included Cartesian readout with fly‐back, TE1/ΔTE/TR = 1.6/1.8/16, flip angle = 15°, cardiac window ~160 ms; key parameters for FF reference included Cartesian readout with fly‐back, TE1/ΔTE/TR = 1.3/2/13.7 ms, flip angle = 5°, cardiac window ~180 ms.

### 2.9 Healthy subjects’ study

In vivo, the same protocols used for the phantom study were used for T2* and FF; whereas MOLLI^46^ (5(3)3 variant) and T2‐GraSE^9^ were considered for T1 and T2, respectively. MOLLI parameters included: TE/TR = 1.4/2.8 ms; FA = 35°; SENSE factor = 2; cardiac window ~220 ms. T2‐GraSE (with black‐blood preparation) key parameters included: nine TEs equally spaced from 9.3 ms to 83.7 ms; FA = 90°; EPI factor = 7; SENSE factor = 3; cardiac window ~85 ms. Sequence parameters for phantom and in vivo are compiled in Supporting Information Table S1, which is available online.

### 2.10 Reconstruction parameters

Dictionary computation was EPG‐based, considering the following values: T1 = [50:20:700, 700:10:900, 900:5:1300, 1300:20:1400, 1400:50:2000] and T2 = [5:5:20, 20:0.5:60, 60:2:100, 100:5:300]. A single TE = 0 was considered, along with fixed B0, B1, and no slice profile correction (enabled by the low flip angles used^48^). After performing an SVD on the MRF dictionary, the ideal compression rank was chosen such that it captured 98% of the matrix energy, resulting in *r* = 8.

Auxiliary cardiac motion‐resolved reconstructions included the following parameters: linearly decreasing soft‐weights ($W_{n}$) up to 50% of the cardiac bin’s width, ADMM iterations = 3, CG iterations = 5, HDPROST self‐similar patches = 20, patch window = 41, λ = 5 × 10^−2^. The first singular image was used for cardiac motion estimation via image registration,^52^ using the following key parameters: cross‐correlation image intensity metric, grid spacing = 3 pixels, bending energy penalty = 0.01, L2 energy penalty = 0.01, number of resolution levels = 5. LRMC reconstructions were performed with the following parameters: ADMM iterations = 6, CG iterations = 5, HDPROST self‐similar patches = 20, patch window = 41, λ = 5 × 10^−3^, *r* = 8. All dense motion fields were cast as sparse matrices ($M_{n}$) considering linear interpolation. Data were also reconstructed with a low‐rank subspace with no motion correction (NMC), by setting $M_{n}=I$. All reconstruction parameters were empirically determined in two representative cases. Reconstructions were performed in a Linux workstation with 12 Intel Xeon X5675 (3.07 GHz) and 200 GB RAM. The proposed approach took ~7:00 (hours:minutes) to reconstruct. Preliminary motion‐resolved images plus motion estimation took ~0:15 minutes, whereas LRMC took ~6:45. Remaining steps (e.g., coil estimation, density compensation function (DCF) computation, water/fat separation and dictionary matching) had negligible costs in time (~ 1 minute). HD‐PROST (denoising step), NiftyReg (image registration step), ESPIRiT (coil estimation), and the non‐uniform Fast Fourier Transform ran as C++ compiled code. All remaining code was implemented in MATLAB (MATLAB R2018b, Natick, Massachusetts: The MathWorks Inc).

### 2.11 Statistical analysis

Phantom measurements were analyzed via normalized RMS error (nRMSE) and fit residuals. In vivo maps were divided into six myocardial segments according to the American Heart Association (AHA),^62^ where the mean value in each segment was considered to evaluate accuracy and the SD in each segment was considered as a surrogate to evaluate precision (also referred to as the spatial variability within the segment). Two‐tailed Student’s t‐test (considering *p* < 0.05 as significant) was used to evaluate statistically significant differences.

Data and code will be made available from the authors upon reasonable request.

## 3 RESULTS

### 3.1 Phantom study

**FIGURE 2:**
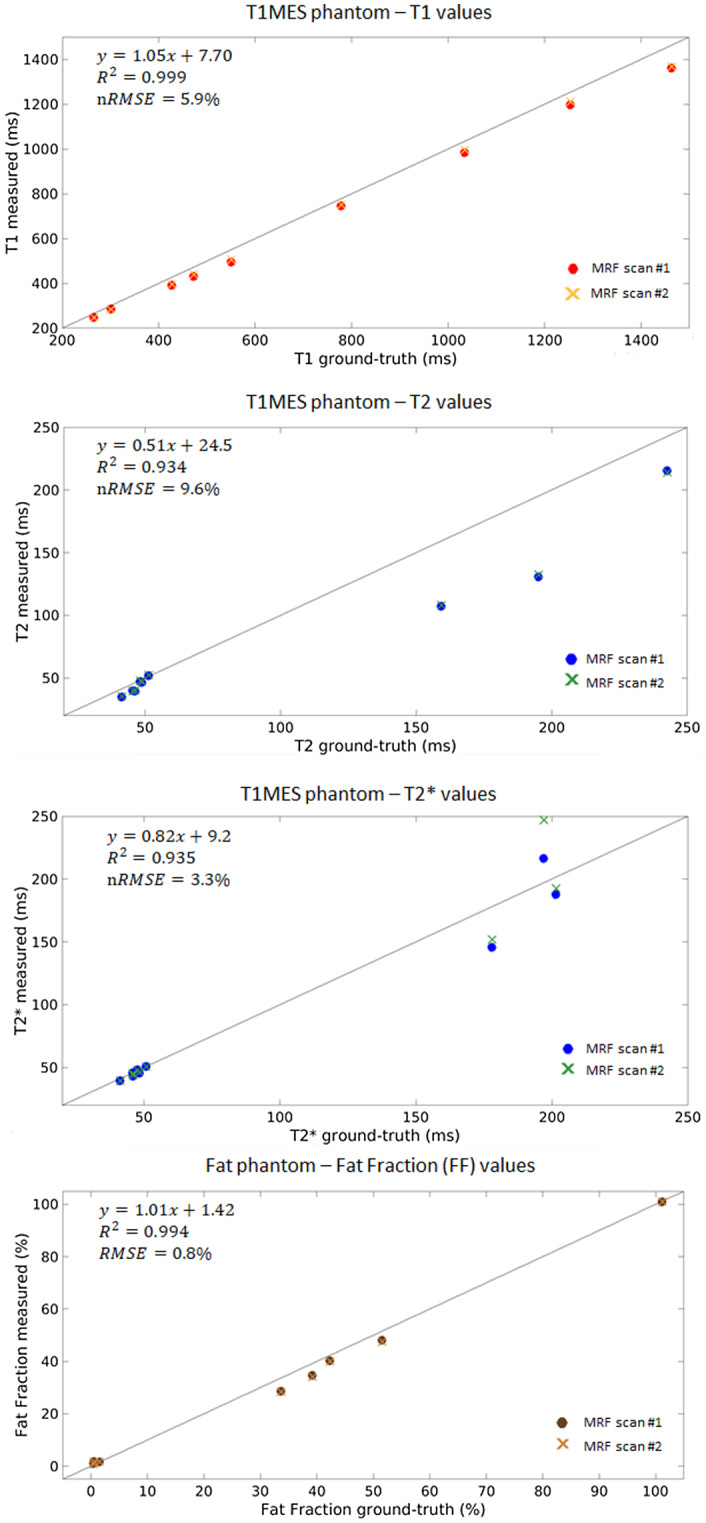
Plots of the mean values within vials for the T1MES and custom fat phantom obtained with the proposed cardiac MRF approach, relative to corresponding references: T1, multi‐echo spin echo (T2), eight‐echo GRE (T2*), and six‐echo GRE (FF). All parameters are in general agreement with the references; however, T2 and T2* values are slightly underestimated within the range of interest (0–100 ms)

T1 values with the proposed cardiac MRF framework were in general agreement despite a small underestimation relative to the IRSE reference (nRMSE = 5.9%). T2 values were underestimated relative to MESE, in the range of interest (0–100 ms) with a negative bias of −3.6 ms (nRMSE = 9.6%); larger errors were observed for very large T2 values, which are not encoded with the proposed cardiac MRF sequence. T2* values presented similar behaviour as T2, with a slight underestimation of −1.1ms (nRMSE = 3.3%) in the range on interest (0–100 ms). FF values were in general agreement with the reference six‐echo GRE, presenting only a small underestimation (RMSE = 0.8%). Both MRF phantom scans were highly repeatable, producing relative differences of 1.4%, 1.2%, 2.2% for T1, T2, and T2*, respectively, and a difference of 0.4% for FF (Figure 2) between two scans performed in the same session after table repositioning. Corresponding values for each vial are reported in Table 1.

### 3.2 Healthy subjects’ study

**FIGURE 3:**
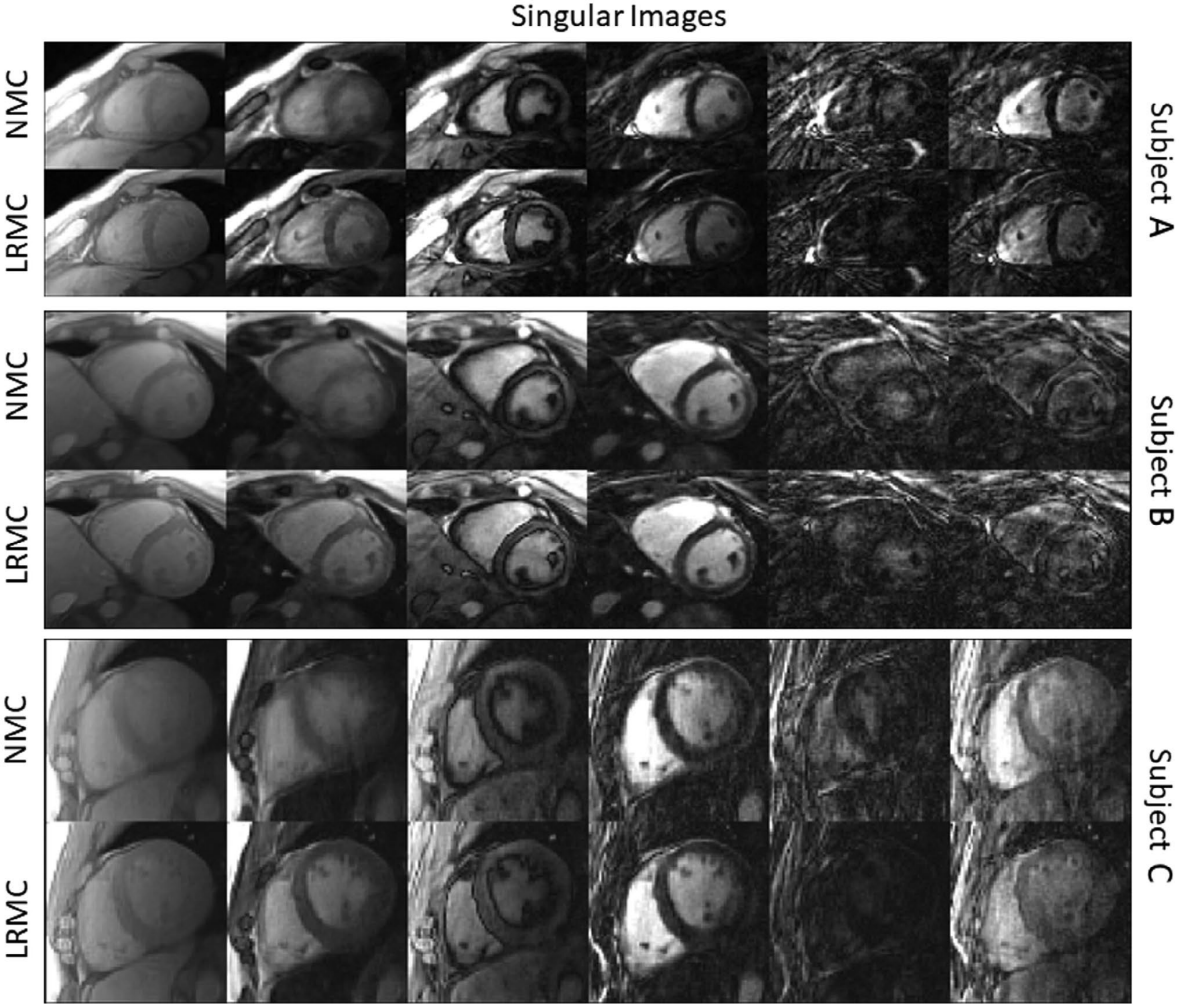
Singular images (first echo) for MRF reconstructed with NMC and with the proposed LRMC for three representative subjects. Considerable blurring artifacts appear in the singular images when motion is not accounted for; these are substantially reduced with LRMC

**FIGURE 4:**
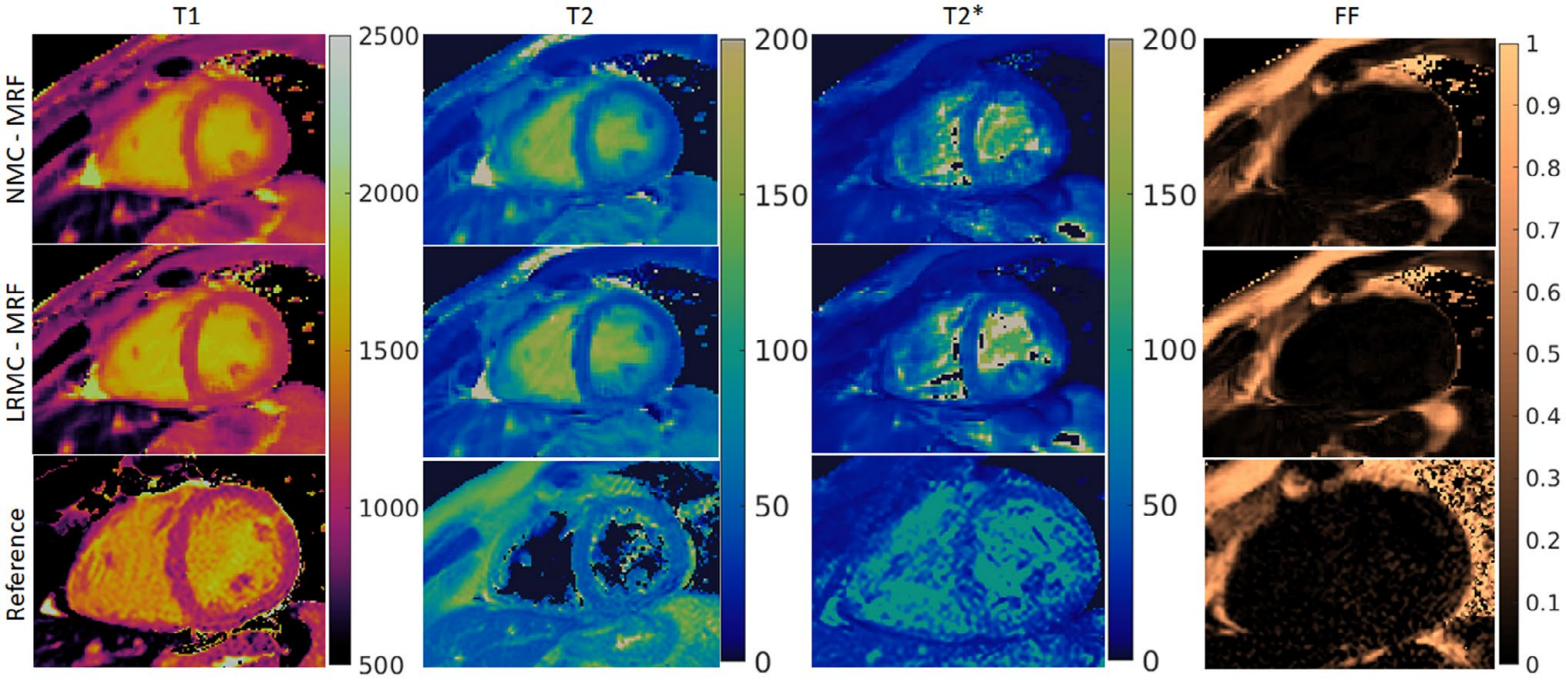
T1, T2, T2*, and FF maps for subject A obtained with NMC‐MRF, the proposed LRMC cardiac MRF (LRMC‐MRF) approach, and the corresponding references: MOLLI, T2‐GraSE, eight‐echo GRE, and six‐echo GRE. With NMC‐MRF, cardiac motion artifacts are observed in the myocardium, primarily for T1 and T2 (less for T2*) with blurring also appearing in the epicardial fat (FF). These artifacts are considerably reduced with the proposed LRMC‐MRF approach, resulting in maps of similar quality to the conventional methods. Co‐registered T1, T2, T2*, and FF maps are intrinsically obtained with the proposed cardiac MRF approach, different to the reference maps acquired in sequential acquisitions

**FIGURE 5:**
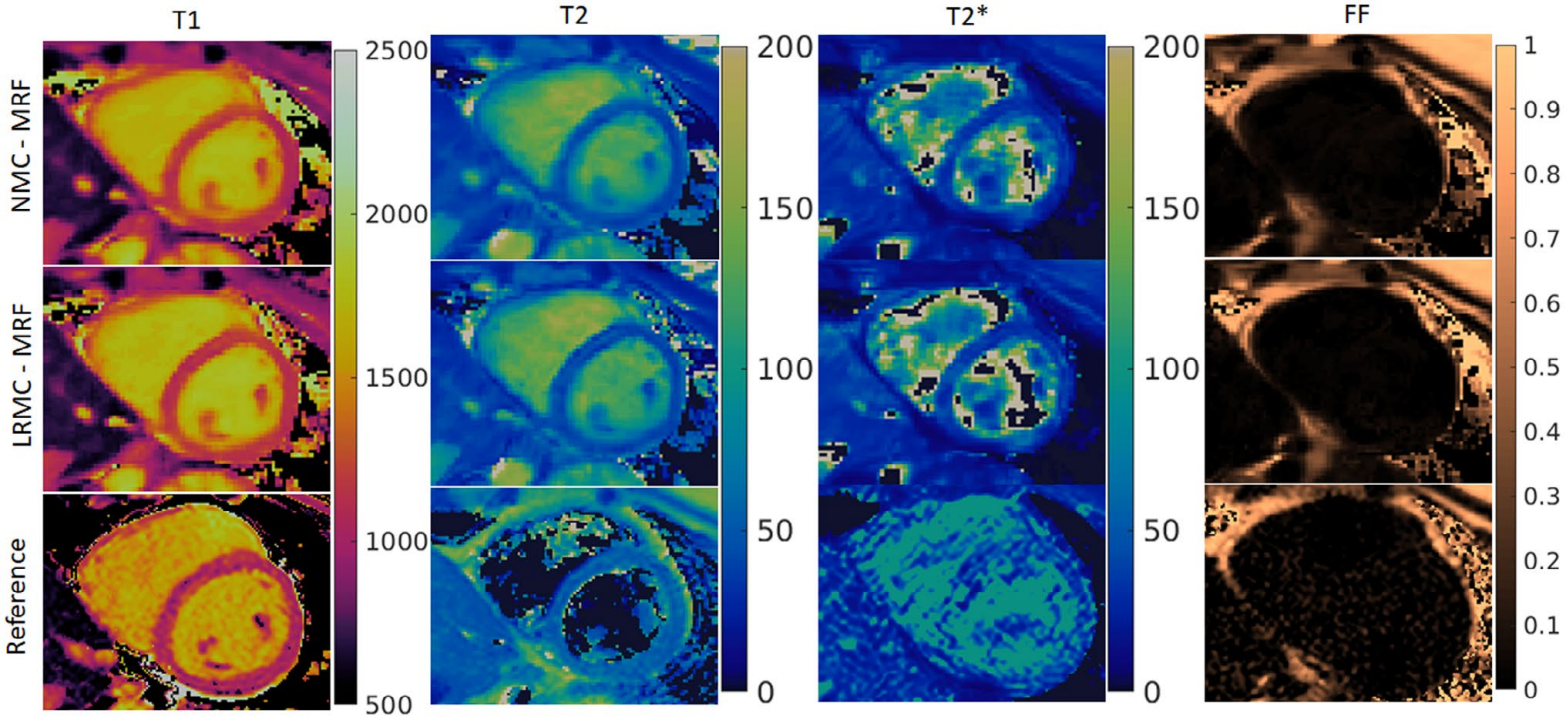
T1, T2, T2*, and FF maps for subject B obtained with NMC‐MRF, the proposed LRMC cardiac MRF (LRMC‐MRF), and the corresponding references: MOLLI, T2‐GraSE, eight‐echo GRE, and six‐echo GRE. With NMC‐MRF, cardiac motion artifacts are observed in the myocardium, primarily for T1 and T2 (less for T2*) with blurring also appearing in the epicardial fat (FF). These artifacts are considerably reduced with the proposed LRMC‐MRF approach, resulting in maps of similar quality to the conventional methods. Co‐registered T1, T2, T2*, and FF maps are intrinsically obtained with the proposed cardiac MRF approach, different to the reference maps acquired in sequential acquisitions

**FIGURE 6:**
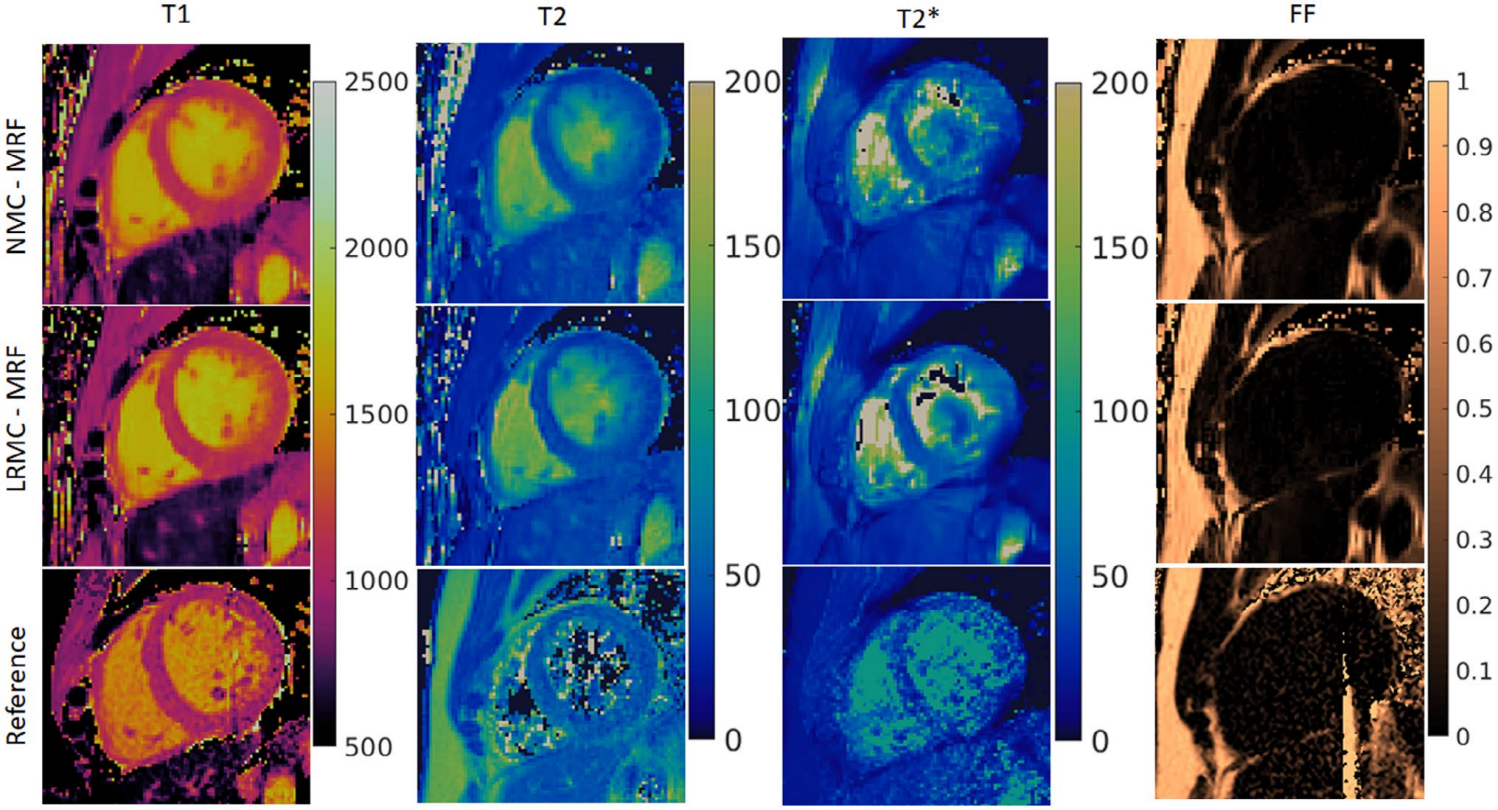
T1, T2, T2*, and FF maps for subject C obtained with NMC‐MRF, the proposed LRMC cardiac MRF (LRMC‐MRF), and the corresponding references: MOLLI, T2‐GraSE, eight‐echo GRE, and six‐echo GRE. With NMC‐MRF, cardiac motion artifacts are observed in the myocardium, primarily for T1 and T2 (less for T2*) with blurring also appearing in the epicardial fat (FF). These artifacts are considerably reduced with the proposed LRMC‐MRF approach, resulting in maps of similar quality to the conventional methods. Co‐registered T1, T2, T2*, and FF maps are intrinsically obtained with the proposed cardiac MRF approach, different to the reference maps acquired in sequential acquisitions

When inspecting in vivo singular images reconstruction with (LRMC) and without (NMC) motion correction, cardiac‐induced motion artifacts due to the extended cardiac acquisition window are evident (Figure 3). For NMC, blurring artifacts are predominant in the first four singular images where most of the cardiac structure appears intact; with LRMC the majority of these artifacts are removed, recovering not only the myocardium wall but also papillary muscles and other small structures. These motion artifacts propagate into the resulting parametric maps and are visible to different degrees in the T1, T2, T2*, and FF maps (Figures 4, 5, 6). Blurring artifacts, primarily in the myocardial wall and papillary muscles, are seen in the T1 and T2 maps with NMC, whereas these artifacts are considerably reduced with LRMC. The quality of the proposed T1 and T2 cardiac MRF maps is similar to conventional MOLLI and T2‐GraSE, respectively. Residual motion artifacts were less obvious in the T2* maps but can still be appreciated in several regions of the myocardium like the septal wall. Visually, the proposed cardiac MRF approach produced substantially less noisy T2* measurements than the conventional eight‐echo GRE reference. For FF, blurring artifacts with NMC were considerable when looking at the epicardial fat; with LRMC the delineation of the fat content is made clearer and its borders with water tissue are sharper. Three additional subjects are shown in Supporting Information Figures S1–S3, presenting similar results.

**FIGURE 7:**
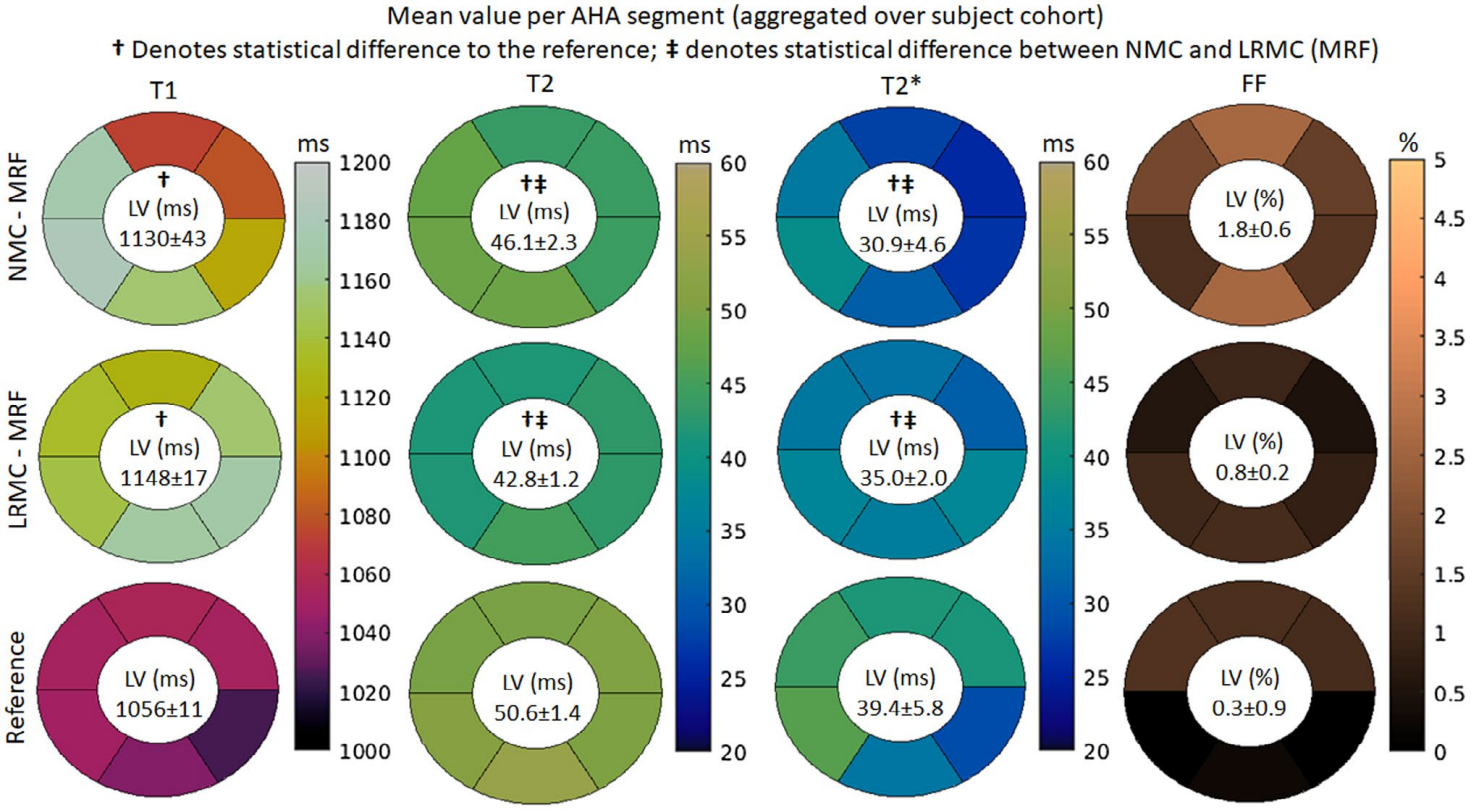
T1, T2, T2*, and FF AHA segmental analysis for the mean values measured with different regions of the left ventricle, obtained with NMC‐MRF, the proposed LRMC cardiac MRF (LRMC‐MRF) approach, and the corresponding conventional methods: MOLLI, T2‐GraSE, eight‐echo GRE, and six‐echo GRE. The proposed LRMC‐MRF estimates higher T1 values than MOLLI, lower T2 and T2* values than T2‐GraSE, and eight‐echo GRE (respectively), and slightly higher FF values than six‐echo GRE. The proposed LRMC‐MRF achieved spatial variability similar or better than conventional methods, and consistently better than NMC‐MRF

Segmental AHA analysis on the mean values of the parameter maps generally revealed reduced left‐ventricular spatial variability for the proposed cardiac LRMC‐MRF approach compared to NMC‐MRF and the corresponding reference maps (Figure 7). Both NMC‐MRF (1130 ms) and the proposed cardiac LRC‐MRF approach (1148 ms) produced higher T1 values than MOLLI (1056 ms); moreover, NMC‐MRF (43 ms) demonstrated higher left‐ventricular spatial variability than LRMC‐MRF (17 ms) and MOLLI (11 ms). NMC‐MRF (46.1 ms) and LRMC‐MRF (42.8 ms) produced lower T2 values than T2‐GraSE (50.6 ms); once again, NMC‐MRF (2.3 ms) had higher segment variability than LRMC‐MRF (1.2 ms) and T2‐GraSE (1.4 ms). Similarly for T2*, both NMC‐MRF (30.9 ms) and LRMC‐MRF (35.0 ms) underestimated compared to eight‐echo GRE (39.4 ms); however, in this case, LRMC‐MRF (2.0 ms) achieved a notably lower segmental variability than NMC‐MRF (4.6 ms) and eight‐echo GRE (5.8 ms). Finally, NMC‐MRF (1.8%) measured a higher FF value than LRMC‐MRF (0.8%) and six‐echo GRE (0.3%); both the NMC‐MRF (0.6%) and the reference six‐echo GRE (0.9%) had higher left ventricular spatial variability than LRMC‐MRF (0.2%). Both NMC‐MRF and LRMC‐MRF measured significantly different values in T1, T2, and T2* (relative to the corresponding references). Furthermore, LRMC‐MRF presented significantly different values in both T2 and T2*.

**FIGURE 8:**
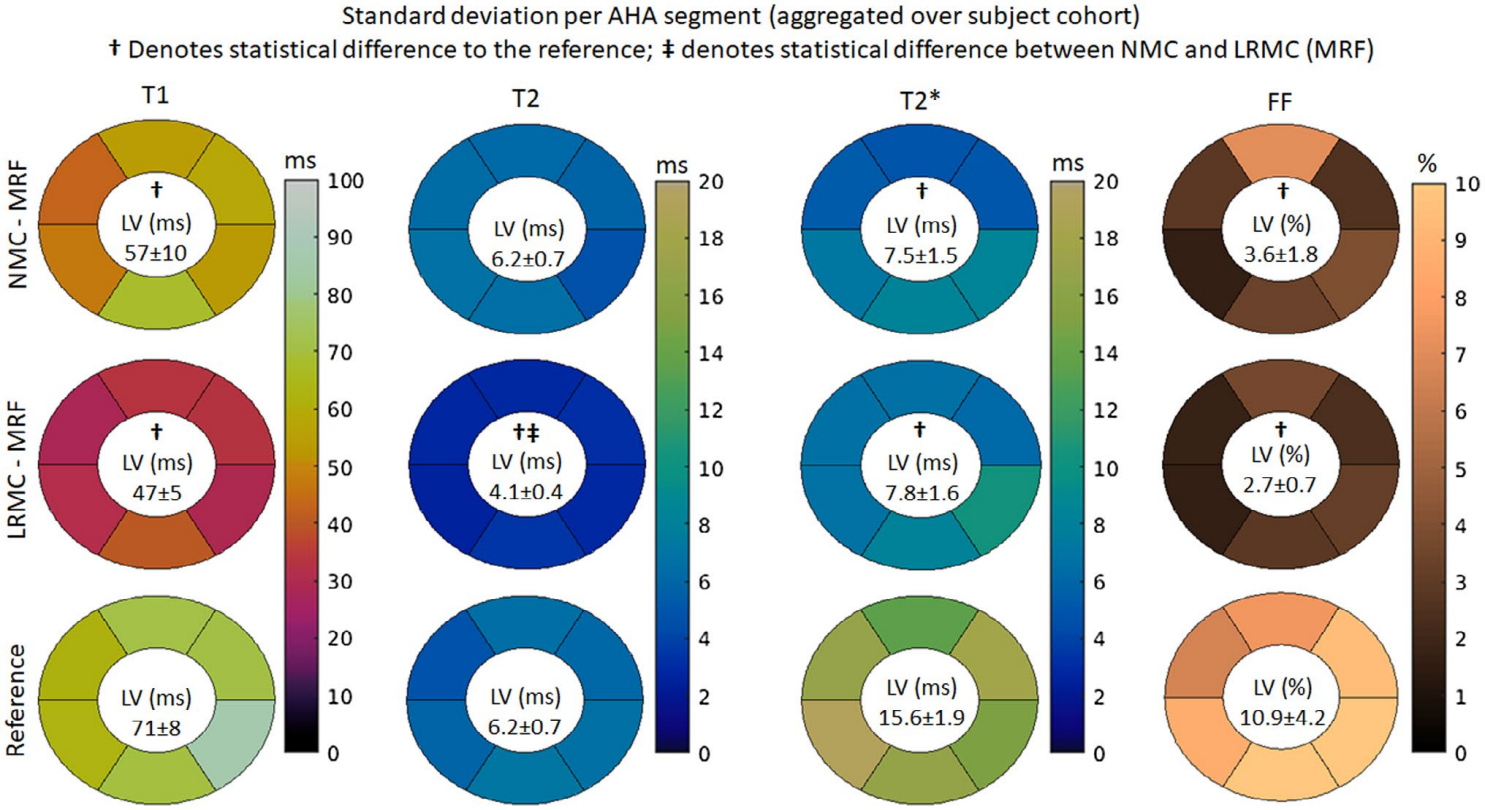
T1, T2, T2*, and FF AHA segmental analysis for the SD measured with different regions of the left ventricle (as a surrogate for precision), obtained with NMC‐MRF, the proposed LRMC cardiac MRF (LRMC‐MRF) approach, and the corresponding conventional methods: MOLLI, T2‐GraSE, eight‐echo GRE, and six‐echo GRE. The proposed LRMC‐MRF consistently obtained higher precision than the conventional methods

A similar AHA analysis was performed considering the SD within segments as a surrogate for precision (Figure 8). Higher SDs were observed for MOLLI (71 ms) relative to NMC‐MRF (57 ms) and LRMC‐MRF (47 ms). T2‐GraSE (6.2 ms) and NMC‐MRF (6.2 ms) presented similar SDs in T2, higher than LRMC‐MRF (4.1 ms). Considerably higher SDs for T2* were observed with eight‐echo GRE (15.6 ms) relative to NMC‐MRF (7.5 ms) and LRMC‐MRF (7.8 ms). Similarly, six‐echo GRE (10.9%) had considerably higher SDs in FF than NMC‐MRF (3.8%) and LRMC‐MRF (2.7%). LRMC‐MRF had significantly lower SDs than the references for all parameters, as did NMC‐MRF with the exception of T2. With the exception of T2, no significant differences were observed between NMC‐MRF and LRMC‐MRF.

**FIGURE 9:**
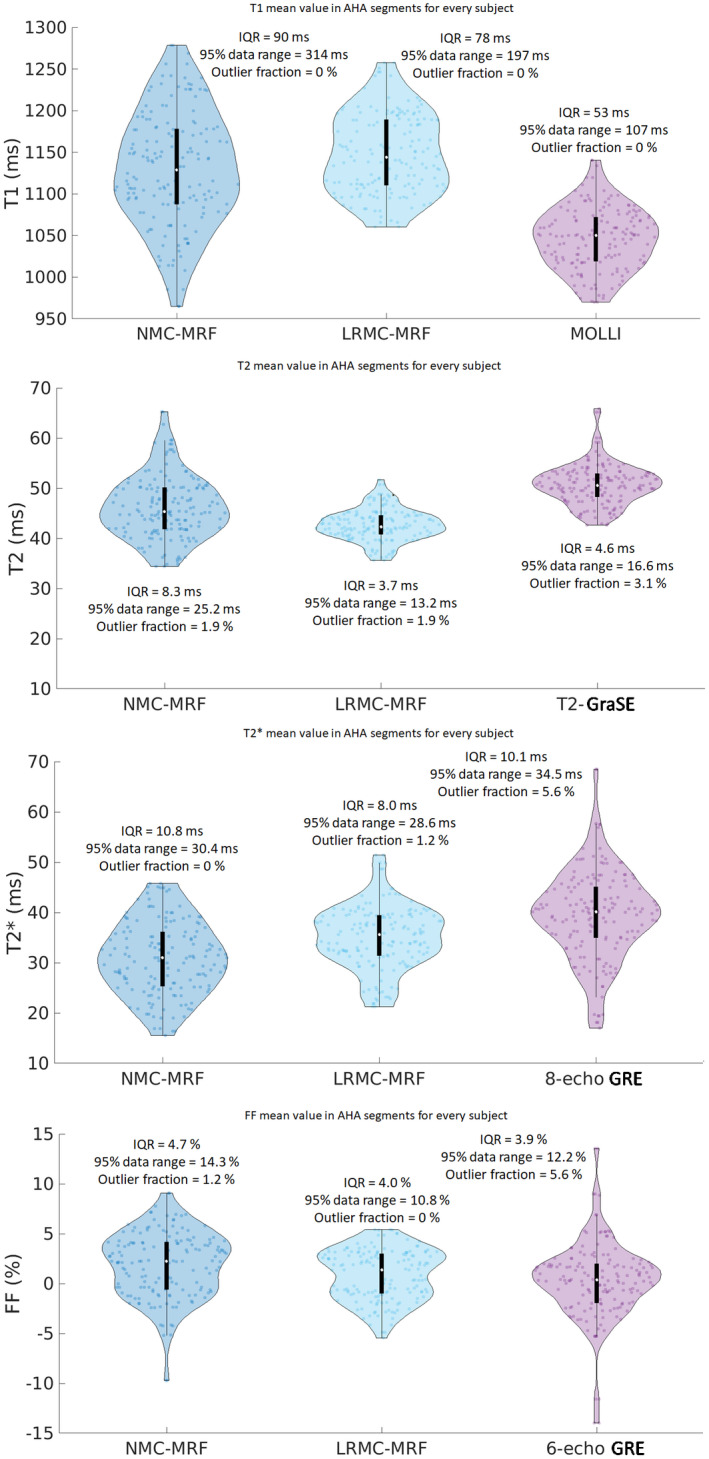
Violin plots for the mean T1, T2, T2*, and FF for all segments and all healthy subjects, obtained with NMC‐MRF, the proposed LRMC cardiac MRF (LRMC‐MRF) approach, and the corresponding conventional methods: MOLLI, T2‐GraSE, eight‐echo GRE, and six‐echo GRE. Each point corresponds to the mean value in a given segment, for a given subject. IQR, 95% data range, and the percentage of segments outside the 95% data range (outlier fraction) are reported for each method. The proposed LRMC‐MRF approach generally presented more compact distributions with less outliers, smaller confident intervals, and IQRs

**FIGURE 10:**
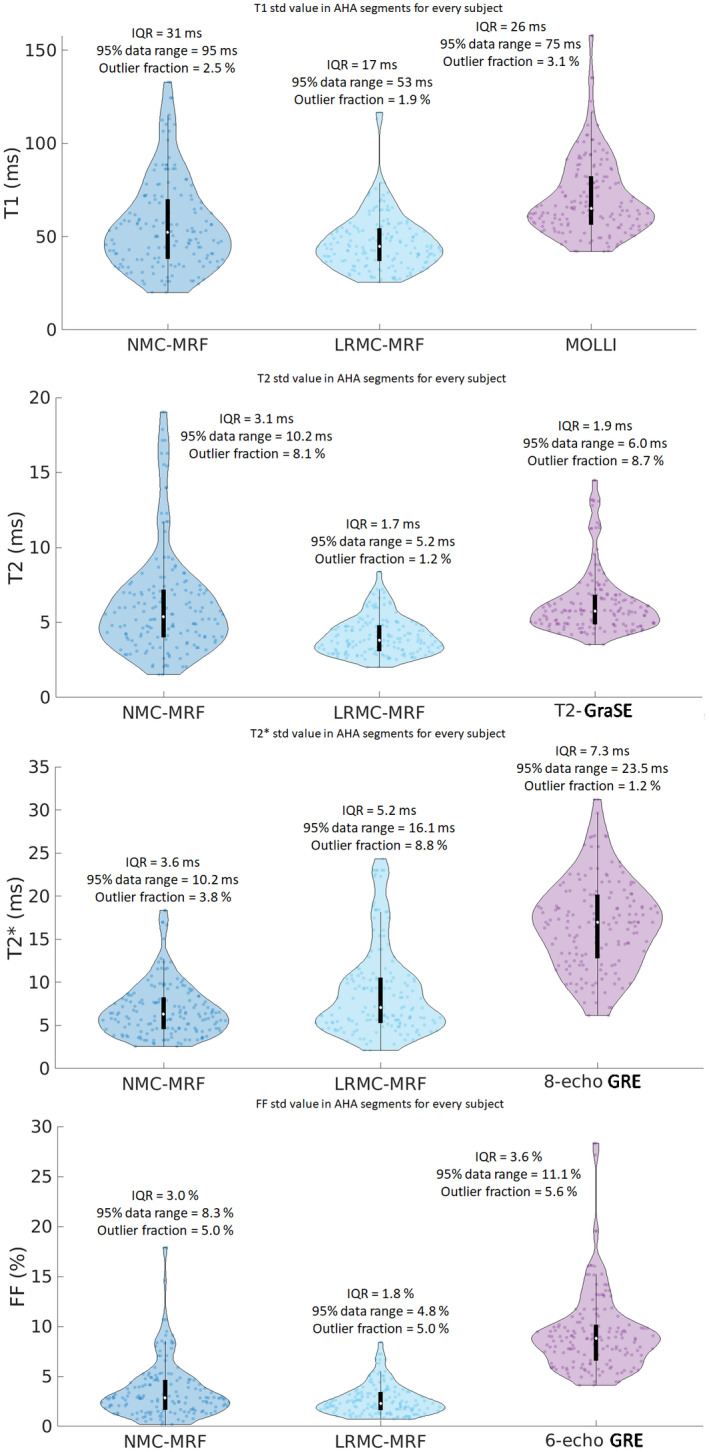
Violin plots for the SD (within each segment) T1, T2, T2*, and FF for all segments and all healthy subjects, obtained with NMC‐MRF, the proposed LRMC cardiac MRF (LRMC‐MRF) approach, and the corresponding conventional methods: MOLLI, T2‐GraSE, eight‐echo GRE, and six‐echo GRE. Each point corresponds to the SD of a given segment, for a given subject. IQR, 95% data range, and the percentage of segments outside the 95% data range (outlier fraction) are reported for each method. In addition to achieving lower (median) SDs, the proposed LRMC‐MRF approach generally presented more compact distributions with less outliers, smaller confident intervals, and IQRs (with the small exception of T2*)

The distributions of the measured parameters in segments across every subject are further characterized in the violin plots of Figures 9 and 10, along with the interquartile range (IQR), the 95% data range of the distributions and the percentage of segments outside the 95% data range (outlier fraction). A reduction in outlier measurements, reduced range of the interquartile, and 95% data range are generally observed with the proposed motion corrected cardiac MRF approach for the mean segmental values (Figure 9). As healthy myocardium is expected to have homogenous values over the left ventricle, these values indicate increased robustness with LRMC‐MRF. When inspecting the spatial variability of all segments (using the SD as a surrogate for precision), once again we observe that LRMC‐MRF generally achieves tighter distributions according to the aforementioned metrics. These metrics suggest that smaller and more predictable errors can be expected with LRMC‐MRF, further contributing to robustness.

## 4 DISCUSSION

In this work, we develop a novel cardiac MRF framework for simultaneous intrinsically co‐registered T1, T2, T2*, and FF mapping in a cardiac triggered, breath‐held, 18 heartbeat scan. An LRMC reconstruction is used to cope with high undersampling factors and cardiac motion in the expanded (480 ms) cardiac acquisition window is used. Low‐rank subspace reconstructions have been investigated for several applications in the field, exploiting temporally redundant information to enable high acceleration factors.^30^, ^31^, ^33^, ^34^, ^35^, ^37^, ^63^, ^64^, ^65^ These approaches are particularly useful in cardiac MRF, where very high acceleration factors are required. Compared to low‐rank, a zero‐filled MRF can produce residual coherent artifacts (especially at higher undersampling factors), which may propagate into errors the parametric maps, as shown in Supporting Information Figures S4 and S5. Errors in the subspace model can also lead to errors in the parameter maps. For example, if only the first half of the proposed sequence (containing T1 encoding: IR‐only) is used to create the subspace, considerable errors will appear in T2. Conversely, if only the second half is considered (T2 encoding: T2p only), then both maps sustain errors (Supporting Information Figure S6). In this case, errors induced in T1 propagate back to T2, which has been described previously.^66^ Simultaneously encoding multiple parameters in a single (ECG‐triggered) breath‐hold is challenging; however, in this study, the cardiac scan efficiency is increased by motion correcting data acquired in a longer cardiac window. This long acquisition window increases scan efficiency, allowing the mapping of additional parameters to remain under a feasible breath‐hold. Parameter mapping is performed by two separate mechanisms: (1) T2* and FF mapping is performed via a graph cut algorithm^58^ together with water/fat separation and (2) the resulting water and fat images are used to estimate T1 and T2 via conventional MRF dictionary matching. A recent study has proposed a method for simultaneous T1, T2, and T2* mapping via conventional strategies (i.e., steady‐state model and exponential fits),^67^ using saturation pulses, T2preps, and five‐echo GRE. To our knowledge, the proposed framework is the first method to simultaneously map T1, T2, T2*, and FF in the heart. Acquisition and reconstruction of four complementary, co‐registered quantitative maps in a single scan may provide valuable diagnostic information for myocardial tissue characterization. Moreover, by resolving the FF the proposed approach produces water‐specific T1 and T2 values, therefore avoiding potential bias from fat.^68^ Although not shown here, we have demonstrated this property in previous studies.^26^, ^57^ Moreover, this framework is expected to also enable post‐contrast ECV mapping (thus producing all SCMR recommended parameters for myocardial tissue characterization with a single sequence), as shown in our previous work.^69^

Phantom experiments demonstrated good agreement between MRF and reference measurements for T1 and FF; however, a negative bias was observed for T2 and T2*. Errors in large T2 values are partly due to the insufficient T2 encoding in this region of the spectrum; however, this bias could also be due to diffusion,^70^ magnetization transfer,^71^ or simplified modeling of the T2 preparation pulse. T2 underestimation in cardiac MRF has been reported in multiple studies.^22^, ^23^, ^26^ The proposed sequence achieved low nRMSE of 5.9%, 3.3%, and 0.8% for T1, T2*, and FF, respectively; a slightly higher nRMSE of 9.6% was observed in T2, corresponding to a bias of −3.6 ms in the range of interest (0–100 ms). The MRF sequence used demonstrated high repeatability between two separate scans, with relative differences <3% for all parameters.

In vivo experiments demonstrated that a considerable amount of cardiac motion occurs in the acquired cardiac window of 480 ms, producing blurring artifacts in the parametric maps if motion is not accounted for. When compared to NMC, the LRMC consistently corrected for motion artifacts leading to a clearer definition of the left ventricle myocardial borders, papillary muscles, right ventricle, and epicardial fat. AHA segmental analysis revealed that the proposed LRMC cardiac MRF produced higher values than conventional MOLLI for T1, lower values than T2‐GraSE for T2, lower values than eight‐echo GRE for T2*, and slightly higher values than six‐echo GRE for FF. With the exception of FF, all MRF measurements differed significantly from the corresponding references: for T1, MOLLI has a known underestimation bias; for T2 (and T2*), MRF has underestimation biases that have been widely reported in the literature and require further study, as mentioned above. The use of a long cardiac window could introduce a positive bias due to flow, particularly for T1 as the parametric encoding could be altered due to in‐flowing blood within the ~500 ms of acquisition in each heartbeat. Significant differences in T2 and T2* were observed between NMC‐MRF and the proposed LRMC cardiac MRF approach due to motion artifacts in the blood‐myocardial border, which also contributed to a higher inter‐segmental variability and higher intra‐segment SD. The proposed approach obtained lower inter‐segment variability than NMC‐MRF and the corresponding references for all parameters except T1 (MOLLI). Considering intra‐segment SD as a surrogate for precision, the proposed cardiac MRF approach (and NMC‐MRF to a lesser degree) achieved higher precision than the corresponding references, especially for T2* and FF. All MRF SDs were significantly lower than the references, with the exception of T2 NMC‐MRF, which was also significantly higher than T2 LRMC‐MRF. This improvement in apparent precision can be explained due to the regularization used in the MRF reconstructions.

This study has several limitations. The proposed sequence requires 18 heartbeats; although this is generally manageable within a breath‐hold, it may not be the case for every patient and efforts to reduce the breath‐hold duration will be considered. The sequence used was heuristically selected based on previous cardiac MRF studies.^22^, ^23^, ^48^, ^72^ Preliminary experiments (not shown) were evaluated using multiple test sequences; the sequence selected (and used in the manuscript) was chosen in order to achieve non‐inferior parametric map quality relative to conventional single‐parameter approaches, in a single breath‐hold. Higher resolution and/or reduce scan time may be considered in the future; these will contribute to higher noise amplification, which may be tackled with improved regularization. The proposed LRMC achieved parametric maps with similar quality to corresponding references; however, performance may improve using sequences with optimal parametric encoding. Higher scan accelerations (supported by improved reconstruction methods), or free‐breathing alternatives may be considered; however, through‐plane motion may provide a challenge in 2D. Despite 2D (potentially with three slices) being the norm in clinical cardiac mapping, 3D is desirable for complete coverage, which may be critical in detection of focal disease. A resolution of 2 mm × 2 mm was used here; however, higher spatial resolutions are desirable for myocardial tissue characterization. Further work on sequence optimization for more efficient acquisitions or stronger regularizers for more powerful reconstructions may contribute toward this goal. Although MRF measurements were generally in agreement with references, an underestimation in T2 and T2* was observed, which has been reported in previous cardiac MRF studies.^22^, ^23^, ^26^ The MRF physics model should be expanded in the future to account for the −8 ms bias observed for in vivo T2. The motion correction itself is limited to in‐plane motion; therefore, residual through‐plane motion could introduce residual blurring, as well as partially account for some T2 (and T2*) underestimation.^40^, ^73^ This can be resolved by extending this approach to 3D or modeling through‐plane motion into the dictionary computation (e.g., with a moving slice profile in the EPG). LRMC reconstruction requires both prior knowledge of the low‐rank subspace and the motion fields. While the subspace is easily estimated from the MRF dictionary, motion estimation requires additional auxiliary reconstructions and image registration steps which add to the complexity of the framework. Alternatively, motion could be resolved instead of corrected as proposed in Multitasking^74^: in that case, a low‐dimensional motion basis can be derived from minimal training data, without the need for additional intermediate reconstruction and registration steps. Multitasking’s framework models generalized motion as an additional dimension in a low‐rank tensor, which enables an effective use of low‐rank priors due to contrast changes (regardless of motion) while enabling motion‐resolved imaging. That approach contrasts with the proposed LRMC that explicitly models the motion into a low‐rank matrix formulism, producing a single motion corrected state. Only healthy subjects were considered in this initial work. Moreover, no in vivo repeatability or reproducibility was performed, which limits the evaluation of the method’s performance. Future studies in additional healthy subjects and patient cohorts will permit the validation of the method in clinical cases and enable further analysis on the benefit of multi‐parametric T1/T2/T2*/FF for myocardial tissue characterization. Extensions of this work may consider 3D free‐breathing applications, correcting for both cardiac and respiratory motion, as well as improved sequence design or regularization to enable higher spatial resolution. Finally, additional parameters of interest, such as T1ρ, may be incorporated into the existing framework to provide further myocardial tissue characterization.

## 5 CONCLUSIONS

Simultaneous and co‐registered T1, T2, T2*, and FF was achieved in a single 18 heartbeat cardiac MRF framework. An LRMC reconstruction is used to correct cardiac motion in long (480 ms) acquisition windows to improve scan efficiency. The proposed approach was successfully validated in phantoms and healthy subjects. Validation in patients with cardiovascular disease is now warranted.

## Supporting information


**FIGURE S1** T1, T2, T2* and Fat Fraction (FF) maps for subject D obtained with No Motion Corrected MRF (NMC‐MRF), the proposed Low Rank Motion Corrected MRF (LRMC‐MRF) and the corresponding references: MOLLI, T2‐GraSE, 8‐echo GRE and 6‐echo GRE. With NMC‐MRF, cardiac motion artefacts are observed in the myocardium, primarily for T1 and T2 (less for T2*) with blurring also appearing in the epicardial fat. These artefacts are considerably reduced with LRMC‐MRF, resulting in maps of similar quality to the conventional methods
**FIGURE S2** T1, T2, T2* and Fat Fraction (FF) maps for subject E obtained with No Motion Corrected MRF (NMC‐MRF), the proposed Low Rank Motion Corrected MRF (LRMC‐MRF) and the corresponding references: MOLLI, T2‐GraSE, 8‐echo GRE and 6‐echo GRE. With NMC‐MRF, cardiac motion artefacts are observed in the myocardium, primarily for T1 and T2 (less for T2*) with blurring also appearing in the epicardial fat. These artefacts are considerably reduced with LRMC‐MRF, resulting in maps of similar quality to the conventional methods
**FIGURE S3** T1, T2, T2* and Fat Fraction (FF) maps for subject F obtained with No Motion Corrected MRF (NMC‐MRF), the proposed Low Rank Motion Corrected MRF (LRMC‐MRF) and the corresponding references: MOLLI, T2‐GraSE, 8‐echo GRE and 6‐echo GRE. With NMC‐MRF, cardiac motion artefacts are observed in the myocardium, primarily for T1 and T2 (less for T2*) with blurring also appearing in the epicardial fat. These artefacts are considerably reduced with LRMC‐MRF, resulting in maps of similar quality to the conventional methods
**FIGURE S4** T1 maps from one representative in‐vivo subject retrospectively reconstructed using subspace modelled LRI or a zero‐filled reconstruction. Three different retrospective undersampling factors are considered, corresponding to 540, 270 and 180 time‐points (R=1, R=2 and R=3, respectively)
**FIGURE S5** T2 maps from one representative in‐vivo subject retrospectively reconstructed using subspace modelled LRI or a zero‐filled reconstruction. Three different retrospective undersampling factors are considered, corresponding to 540, 270 and 180 timepoints (R=1, R=2 and R=3, respectively)
**FIGURE S6** T1 and T2 maps from one representative in‐vivo subject retrospectively reconstructed using subspace modelled LRI, LRI using only data related to T1 encoding (IR only) and LRI using only data related to T2 encoding (T2p only)
**TABLE S1** Sequence parameters used for phantom and in vivo experiments
