# Supplementary material for: Myocardial T1, T2, T2*, and fat fraction quantification via low‐rank motion‐corrected cardiac MR fingerprinting
Source: Magn Reson Med. 2022 Jan 26;87(6):2757–74. doi: 10.1002/mrm.29171 (PMC9306903; doi:10.1002/mrm.29171)
Supplement: Supplementary file 1 — FIGURE S1 T1, T2, T2* and Fat Fraction (FF) maps for subject D obtained with No Motion Corrected MRF (NMC‐MRF), the proposed Low Rank Motion Corrected MRF (LRMC‐MRF) and the corresponding references: MOLLI, T2‐GraSE, 8‐echo GRE and 6‐echo GRE. With NMC‐MRF, cardiac motion artefacts are observed in the myocardium, primarily for T1 and T2 (less for T2*) with blurring also appearing in the epicardial fat. These artefacts are considerably reduced with LRMC‐MRF, resulting in maps of similar quality to the conventional methods FIGURE S2 T1, T2, T2* and Fat Fraction (FF) maps for subject E obtained with No Motion Corrected MRF (NMC‐MRF), the proposed Low Rank Motion Corrected MRF (LRMC‐MRF) and the corresponding references: MOLLI, T2‐GraSE, 8‐echo GRE and 6‐echo GRE. With NMC‐MRF, cardiac motion artefacts are observed in the myocardium, primarily for T1 and T2 (less for T2*) with blurring also appearing in the epicardial fat. These artefacts are considerably reduced with LRMC‐MRF, resulting in maps of similar quality to the conventional methods FIGURE S3 T1, T2, T2* and Fat Fraction (FF) maps for subject F obtained with No Motion Corrected MRF (NMC‐MRF), the proposed Low Rank Motion Corrected MRF (LRMC‐MRF) and the corresponding references: MOLLI, T2‐GraSE, 8‐echo GRE and 6‐echo GRE. With NMC‐MRF, cardiac motion artefacts are observed in the myocardium, primarily for T1 and T2 (less for T2*) with blurring also appearing in the epicardial fat. These artefacts are considerably reduced with LRMC‐MRF, resulting in maps of similar quality to the conventional methods FIGURE S4 T1 maps from one representative in‐vivo subject retrospectively reconstructed using subspace modelled LRI or a zero‐filled reconstruction. Three different retrospective undersampling factors are considered, corresponding to 540, 270 and 180 time‐points (R=1, R=2 and R=3, respectively) FIGURE S5 T2 maps from one representative in‐vivo subject retrospectively reconstructed using subspace modelled LRI or a ze [file MRM-87-2757-s001.docx]

Supporting Information Table S1.

**Supporting Information Table S1.** Sequence parameters used for phantom and in vivo experiments.

| Phantom  Parameters | IRSE-T1 | T2-MESE | 8-echo GRE | 6-echo GRE | Cardiac MRF |
| --- | --- | --- | --- | --- | --- |
| FOV (mm) | 256x256 | 256x256 | 256x256 | 256x256 | 256x256 |
| Resolution (mm) | 2x2 | 2x2 | 2x2 | 2x2 | 2x2 |
| TE (ms) | 6 | 1.1 | 1.6 | 1.3 | 1.6 |
| ΔTE (ms) | - | - | 1.9 | 2.0 | 1.8 |
| TR (ms) | 10000 | 2.3 | 17 | 14 | 16 |
| FA (º) | 90 | 90 | 15 | 5 | 15 |
| Cardiac window (ms) | - | - | 170 | 180 | 480 |
| Scan time  (at 60 BPM) | 2h20m | 10m | 9s | 7s | 18s |
| *In vivo*  Parameters | **MOLLI** | **T2-GraSE** | **8-echo GRE** | **6-echo GRE** | **Cardiac MRF** |
| FOV (mm) | 312x312 | 312x312 | 312x312 | 312x312 | 256x256 |
| Resolution (mm) | 2x2 | 2x2 | 2x2 | 2x2 | 2x2 |
| TE (ms) | 1.3 | 8.3 | 1.6 | 1.3 | 1.6 |
| ΔTE (ms) | - | 8.3 | 1.9 | 2.0 | 1.8 |
| TR (ms) | 2.6 | 1000 | 17 | 14 | 16 |
| FA (º) | 35 | 90 | 15 | 5 | 15 |
| Cardiac window (ms) | 204 | 80 | 170 | 180 | 480 |
| Scan time  (at 60 BPM) | 11s | 14s | 9s | 7s | 18s |

Supporting Information Figure S1.


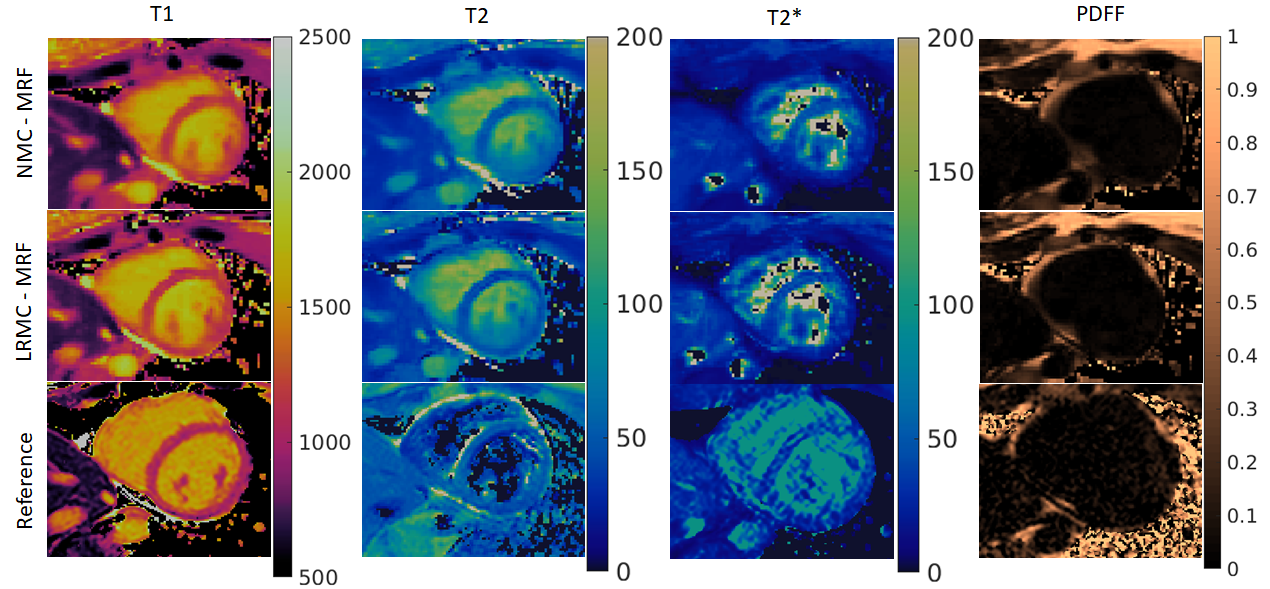


**Supporting Information Figure S1.** T1, T2, T2* and Fat Fraction (FF) maps for subject D obtained with No Motion Corrected MRF (NMC-MRF), the proposed Low Rank Motion Corrected MRF (LRMC-MRF) and the corresponding references: MOLLI, T2-GraSE, 8-echo GRE and 6-echo GRE. With NMC-MRF, cardiac motion artefacts are observed in the myocardium, primarily for T1 and T2 (less for T2*) with blurring also appearing in the epicardial fat. These artefacts are considerably reduced with LRMC-MRF, resulting in maps of similar quality to the conventional methods.

Supporting Information Figure S2.


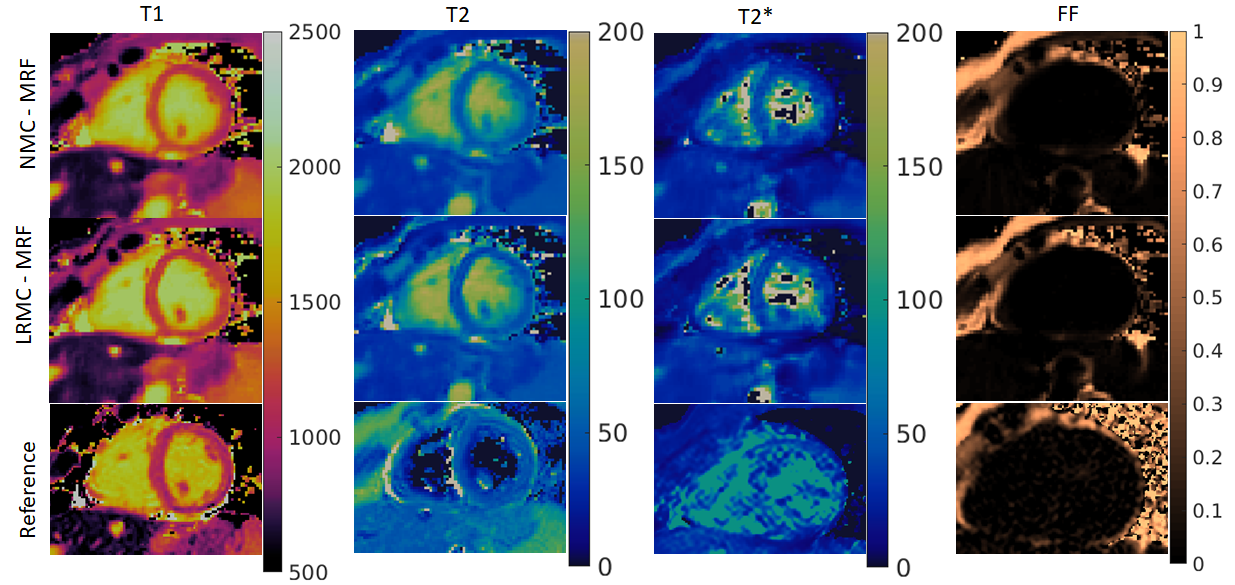


**Supporting Information Figure S2.** T1, T2, T2* and Fat Fraction (FF) maps for subject E obtained with No Motion Corrected MRF (NMC-MRF), the proposed Low Rank Motion Corrected MRF (LRMC-MRF) and the corresponding references: MOLLI, T2-GraSE, 8-echo GRE and 6-echo GRE. With NMC-MRF, cardiac motion artefacts are observed in the myocardium, primarily for T1 and T2 (less for T2*) with blurring also appearing in the epicardial fat. These artefacts are considerably reduced with LRMC-MRF, resulting in maps of similar quality to the conventional methods.


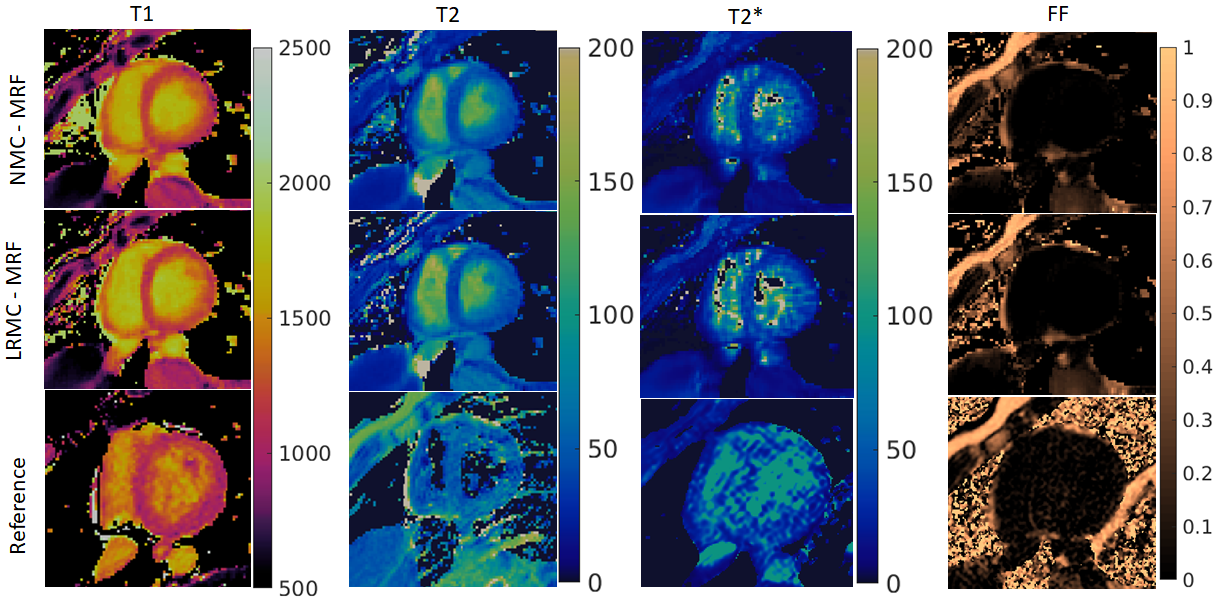
Supporting Information Figure S3.

**Supporting Information Figure S3.** T1, T2, T2* and Fat Fraction (FF) maps for subject F obtained with No Motion Corrected MRF (NMC-MRF), the proposed Low Rank Motion Corrected MRF (LRMC-MRF) and the corresponding references: MOLLI, T2-GraSE, 8-echo GRE and 6-echo GRE. With NMC-MRF, cardiac motion artefacts are observed in the myocardium, primarily for T1 and T2 (less for T2*) with blurring also appearing in the epicardial fat. These artefacts are considerably reduced with LRMC-MRF, resulting in maps of similar quality to the conventional methods.

Supporting Information Figure S4.


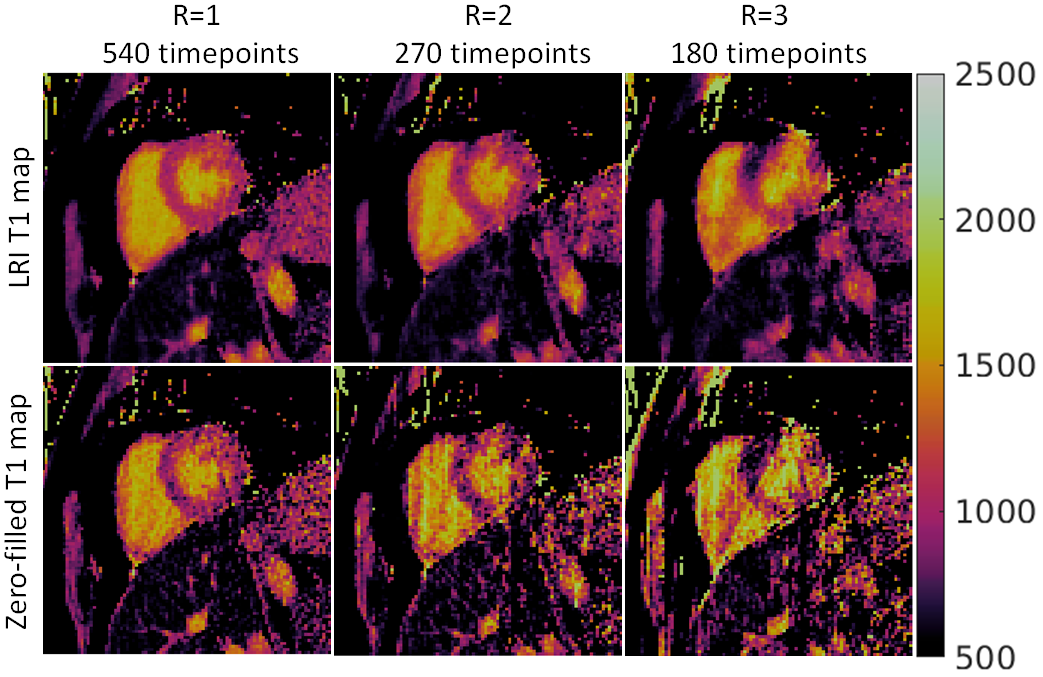


**Supporting Information Figure S4.** T1 maps from one representative in-vivo subject retrospectively reconstructed using subspace modelled LRI or a zero-filled reconstruction. Three different retrospective undersampling factors are considered, corresponding to 540, 270 and 180 time-points (R=1, R=2 and R=3, respectively).

Supporting Information Figure S5.


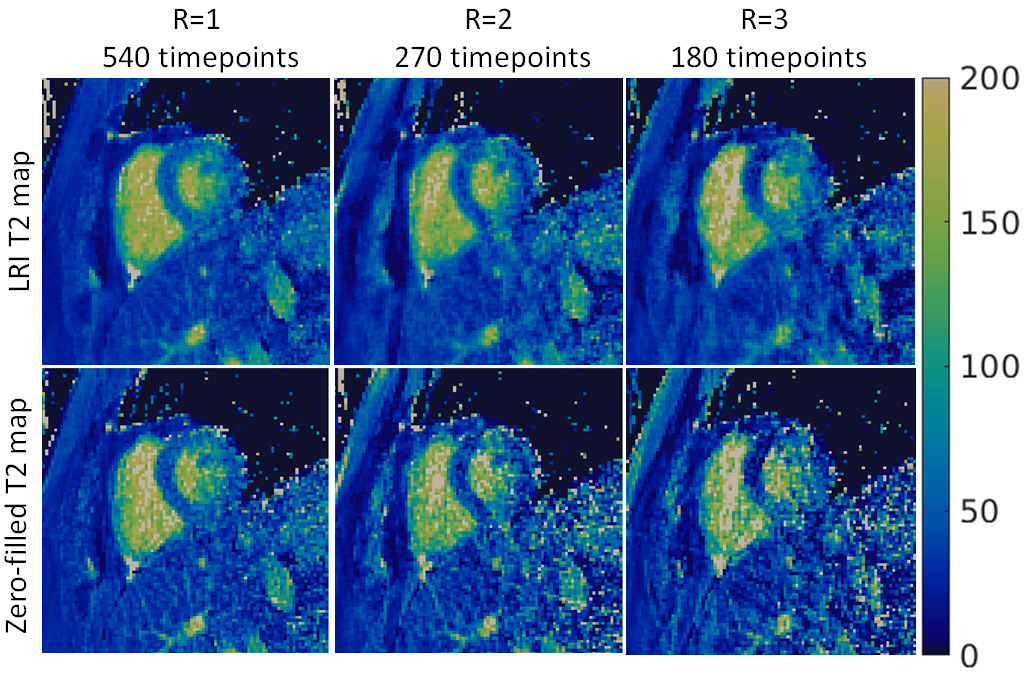


**Supporting Information Figure S5.** T2 maps from one representative in-vivo subject retrospectively reconstructed using subspace modelled LRI or a zero-filled reconstruction. Three different retrospective undersampling factors are considered, corresponding to 540, 270 and 180 timepoints (R=1, R=2 and R=3, respectively).

Supporting Information Figure S6.


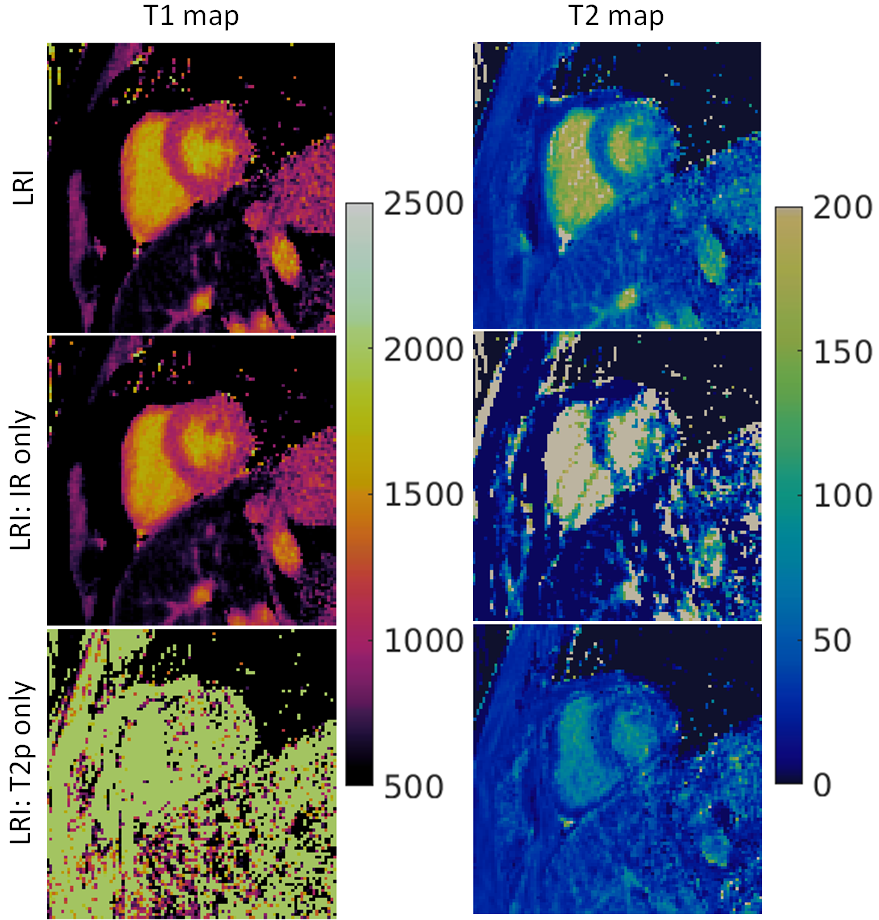


**Supporting Information Figure S6.** T1 and T2 maps from one representative in-vivo subject retrospectively reconstructed using subspace modelled LRI, LRI using only data related to T1 encoding (IR only) and LRI using only data related to T2 encoding (T2p only).
